# Present Tendencies and Future Developments

**Published:** 1913-10-18

**Authors:** 


### NON-PANEL AND PANEL PRACTICE UNDER THE INSURANCE ACT.

### Present Tendencies and Future Developments.

Those who take sufficient interest in medical politics, or in the National Insurance Act, to watch the working-out of the "medical benefit" in so far as it includes medical treatment of the insured, are well aware that the disunion which sundered at the beginning of the year the medical profession, and above all its principal organisation, the British Medical Association, is if anything more profound than it was ten months ago. This statement will, we fancy, be admitted by anyone who reads regularly the *British Medical Journal*, the official organ of the Association; and our own columns have borne equally convincing testimony to the same effect for some little time. We are not concerned just now with the reasons for the hopeless failure of the doctors to stand by each other and by their Association in January. Suffice it that we were frankly astounded by what took place, and that the phenomenon has to be taken into account in any endeavour to foresee the probable conduct and action of the medical profession if ever it finds itself again confronted with a similar crisis.

### Where are We?

The present attitude of the doctors towards the Insurance Act and the panel system may be summed up with considerable confidence; and in what follows an honest attempt is made to give a perfectly impartial account of the situation. A majority of the practising medical men in Britain are on the panels; but a very considerable minority are not and are taking no part in the working of the Act. Of those on the panels, it is safe to say that the great majority went on without enthusiasm; in fact, sorely against their wills. Amongst this large body there is some discontent with various points in the panel system; but, on the whole, this discontent is less than the "panellers" themselves probably anticipated.

What is the explanation of this partial falsification of prophecies? It is just this, that the panellers are, speaking of the general average run, giving their insured patients the same perfunctory attendance and treatment which they formerly gave their "club" patients; and are not only much better paid, but are also freed from the domination of those hard taskmasters and inveterate "sweaters," the officials of the Friendly Societies. As before the passing of the Act, urgent cases and practically all surgical cases are sent to voluntary hospitals, which are obligingly continuing to do the work for the panellers and for the Insurance Committees without a word of thanks or a ha'p'orth of subvention.

All the tall talk about the latest refinements of diagnosis being placed at the service of the insured and about the highly superior quality and quantity of the attendance they would receive has so far meant nothing at all. The reason for this is that to get the panels filled at all (and keep them filled) it has been found necessary to exact nothing more in the way of service from the panellers than that which they were accustomed to give to the Friendly Societies. Conceivably at some future time, when the Act has got into better and more certain working order, the screw will be put on and a higher standard of professional labour will be exacted; but that time has not come yet. It cannot be, indeed we know full well that it is not, congenial to any medical man with reasonably high ethical ideals to give this half-hearted service; but those, and they are very many, who feel thus regard themselves as having been cozened into acceptance of derogatory terms last January, and they say that if they accepted on their lists only such numbers of the insured as they would have time to treat without sacrifice of efficiency, the capitation rate would fail to yield them what for want of a better term may be called a "living wage."

The panellers, then, are moderately satisfied with their present position, and, broadly speaking, the least efficient and least high-minded are the best satisfied with themselves and their servitude. The non-panellers, on the other hand, are as hostile as ever to the Act, and even more hostile than before to the panel system and the panellers. Indeed, the non-panellers, excluding those who are not in general practice, are becoming more and more actively "anti-panellers," and their discontent is being signalised in many different districts by outbursts such as that which has recently taken place in Wandsworth. Leagues, guilds, and several similar societies have sprung into existence, each voicing the objections of a particular sect of anti-panellers.

### The Call for Unity.

The most serious result of this growth of feeling is the accentuation of the deep cleavage in the ranks of the British Medical Association early in the year. The anti-panellers, or some of them, are becoming so incensed against the panellers and so convinced of the political powerlessness of the Association, that they contemplate secession from the Association, and a few of them have individually carried out that intention to actual resignation.

With all respect to the anti-panel organisations and their officers, we believe such a policy of disunion to be a grave strategical error. Granted that the futility of the Association and the general invertebracy of its members were proved to the hilt in January, the British Medical Association remains the most powerful engine at the present moment which the medical profession possesses for concerted and political action. Granted also that so long as most of its members remain on the panels, the anti-panellers cannot hope to control the Association's attitude towards the Act and its workings, yet by remaining members of it they can exert great influence on its decisions and on its members, an influence which in time may go far to leaven the lump. By all means let the anti-panellers, if they think fit, organise apart from the Association for the furtherance of their aims and views; but that is no sufficient reason for their immediate retirement from the British Medical Association.

### The Ultimate Prospect.

So much then for the present state of affairs in respect of panel practice and the position of the medical profession. Dangerous as the rôle of prophecy usually is, it is doubly difficult at present. Nevertheless, there are one or two contingencies of the future which seem tolerably certain to materialise, and their probable effects upon the panel system can be estimated. To take the long view at the present time is not easy, but it is well worth while to make the attempt.

To begin with a point of the more immediate, not the remoter, future, it is to be noted that an exceptionally fine and (for adults) healthy summer has only just come to an end. Consequently, a great many panellers are handling comfortable cheques as payment for by no means an excessive amount of work. When winter comes, however, the percentage of sick among the insured will rise rapidly, and those who have light-heartedly accepted two or three or four or six thousand on their panel lists will find themselves with overflowing surgeries and lengthy visiting lists just at that time of year when the more remunerative private practice is also at its busiest. Indeed, if a good many panellers are going to get any time at all for food, sleep, or recreation, it is certain that they will have to neglect their insured patients, whereby hardship to and grumbling amongst the latter are bound to arise. It will be said that this applies just as much to last January and February as to next November and December. But when medical benefit began many of the insured did not at first take full advantage of it, nor at that time were there any panellers with the swollen lists which some of them now possess.

### 1916.

Let us endeavour to look somewhat further ahead into the future. In two and a half years' time comes the first valuation of the assets and liabilities of the approved societies. Every expert authority is agreed on one point—namely, that this valuation will disclose grave deficiencies; in other words, that the position of the approved societies, or most of them, will be financially perilous. This is the natural result which must flow from the large excess of the claims upon the funds over the actuarial estimates upon which the finance of the Act was based. Now the Act provides that when one of the periodical valuations discloses such a deficiency the Society which finds itself in that unpleasant predicament must reduce correspondingly the benefits which it provides for its members; in other words, it must curtail its expenditure until it is once more solvent. When 1916 comes and the results of the valuations of the different approved societies are made known, there will certainly be trouble. The insured are not likely to be pleased at the prospect of receiving nine, eight, or seven shillings a week sick benefit instead of the ten to which they are at present entitled. The societies in their turn may be trusted to do everything in their power to avoid the inevitable unpleasantness with their members by economising on everything else before reducing sick benefits.

In this dilemma, what way out are they likely to seek? The societies themselves cannot, as the Act stands, reduce the credits for medical attendance and drugs. But they can, and indubitably will, use their enormous political power to secure some such arrangement. If anyone holds this to be a mere bogey or chimera, let him study what has taken place in Germany, from which country Mr. Lloyd George's Act was openly and admittedly copied. In Germany the reduction of the capitation rate for medical attendance upon the insured *has actually taken place.*

### The Parting of the Ways.

Then, if not before, the profession will realise that the parting of the ways has indeed been reached. Then the gulf between paneller and anti-paneller will be fixed once and for all. Then the medical profession will have need of its wisest counsels, its perfectest organisation, its greatest solidarity, if it is not as a learned profession to go under finally and for good. Then the anti-panellers will come by their own, and it is with that date—the spring of 1916—in view that they should conserve their energies and concentrate their forces. The next two and a half years must be devoted to the spade-work which shall prepare the way for the upheaval of 1916. Meanwhile, they can make common cause with one another, abandon a futile policy of individual secession from the British Medical Association, and refrain from forming many separate societies. If they find eventually that, in spite of all, the Association is permanently captured by panellers, then will they be justified in throwing the Association over. Meanwhile they should use the leverage of their collected and united prospective resignations to forward their principles.

This forecast of the future crisis which may, and we believe will, confront the medical profession depends on two events: one, the disclosures of serious deficiencies in the assets of the approved societies, and the other, successful pressure by the latter upon the Government of the day to economise on medical benefit as well as on sick benefit. Either of those forecasts may prove wrong; we hope they will. But the signs of the times, in so far as we can read them, make them both possible, and we fear even probable. Such a *dénouement* offers, in our judgment, the main prospect of success for the anti-panel party, and if they are wise their plans will be laid accordingly. The panellers are now making hay while the sun shines, but the day may come when their glorious summer turns into a winter of discontent—not to say a bitter frost.

